# Benefit of Clergy

**Published:** 1909-12-11

**Authors:** 


### The Hospital

A Journal of

The Medical Sciences and Hospital Administration.

New Series. No. 146, Vol. VI. [No. 1218, Vol. XLVII.] SATURDAY, DECEMBER 11, 1909.

### Benefit of Clergy.

A mild flutter was recently created by an announcement from the rector of an Episcopalian Church in New York that he intended formally to devote himself to the healing of the sick. It is a sign of the times, and one not without interest, to find the orthodox clergy setting out upon the rocky road already trodden, but by no means smoothed, by many thousands of Christian Scientists. The originator of the movement appears to have been the Rev. Dr. Worcester, of Boston; but, as we have said, New York has followed suit, and not very long ago the attractiveness of the idea was evinced by a meeting of Anglican clergy and laymen, held in England, to form a Central Church and Medical Union to consider the subject of spiritual healing. The central idea of the new movement is based upon the supposition, by no means baseless nor by any means new, that the stimulus of faith, among many other obscure mental stimuli, is capable of modifying the functions of the body. This being the case, it is held by the promoters of the new movement that, if medicine and the church were to combine forces, the combination would result in a great accretion of therapeutic strength; and that the healing power of faith, thus regularised and given a more accurate direction, might be expected to bear better and more abundant fruit than if it were left to the random exploitation of Eddyites and other fanatics of that order. No one will deny that there is much of truth in this proposition. Although any new mystic cult seems always able to command a large hearing, the spread of Christian Science would never have advanced so fast as it undoubtedly has were there not something of justice in its claims, however deeply the truth in it may be overlaid by senseless superstition and follies; and if it were possible to systematise what is valuable in the practice of "mental healing" and have done for good with the trashy growth of imposture and bombast which encompasses the business, no one would be better pleased than the medical practitioner. For, much as we have advanced in the treatment of organic maladies, we must all of us feel, and that not seldom, a painful sense of our deficiencies in the treatment of functional troubles, and it is to diseases of this order that projectors of the new movement intend to devote themselves.

The present-day methods of treating disorders of function from the mental, as opposed to the physical, side fall into two broad groups. The one includes the practice of formal suggestion, the other —the "rational psychotherapy" of continental writers—implies an attempt to guard the victim of functional disease from the effects of his own fears and auto-suggestions by a direct appeal to his reason; by explaining to him the influence exerted by mental impressions upon the workings of the body, and by putting him into a position to secure that the influences acting upon the mind shall be as favourable as possible, instead of the reverse, as is too commonly the case with such people. It is clear—we speak with all reverence—that the invocation of religious fervour as a curative agent in connection with functional disease falls into the former group, the group of treatments essentially suggestive. Now, although no sensible man will deny the power of suggestion in such cases, it remains the fact that treatment by this means is steadily declining in favour. It has always been looked upon with suspicion, a suspicion bred by the unconscionable rogueries to which it lends itself; but it has, nevertheless, had a good trial at the hands of reputable members of our profession. The chief theoretical objection may be stated as follows: It is a general, though not universal, characteristic of the neurotic class who are the sufferers from functional diseases, that they are deficient in rational sense, over-susceptible to emotional stimuli which they allow to operate without due control by the reason, and easily persuaded, not only by the suggestions of others, but by auto-suggestions excited by the physical vagaries to which their economy is subject. This being the case, it seems clear that the best way to protect them against this inherent disability is to cultivate in them a critical attitude, a habit of submitting all their conclusions to the test of reason before acting upon them. This is the aim of "rational psychotherapy." The effect of treatment by bald suggestion, whether religious or other, is totally contrary to this principle. It acts by replacing an evil suggestion with a good one, but the good suggestion, when based upon a religious belief, is a mere act of faith, and thus *ex hypothesi* the antithesis of an act of reason. It is perfectly true that in a number of cases the victims of functional disease are inherently so deficient in rational sense that appeals to reason are made to them in vain. In these cases, since an attempt must be made to achieve an improvement, even though it be but temporary, the use of pure suggestion appears justified. But it must be remembered how liable are such people to religious obsessions, the development of which leaves the victim in a worse condition than before. Without, therefore, condemning the new movement out of hand, we may conclude that its path is full of dangers and that its success or failure will turn upon the special fitness or unfitness of the individuals who put its teachings into practice. The devout believer who yet commands a due sense of proportion, who can temper the fervour of his beliefs by a just appreciation of the mental requirements and dangers of the subject to whom he addresses himself, may do great good. But to legitimise the practice of mental healing by men whose chief claim to fitness for the task lies in unbounded religious enthusiasm involves so many risks that the churches will do well to observe the greatest caution in officially undertaking the business.

